# New Insights into the Mechanisms of Gene Electrotransfer – Experimental and Theoretical Analysis

**DOI:** 10.1038/srep09132

**Published:** 2015-03-16

**Authors:** Mojca Pavlin, Maša Kandušer

**Affiliations:** 1 Faculty of Electrical Engineering, University of Ljubljana, Tržaška 25, 1000 Ljubljana, Slovenia

## Abstract

Gene electrotransfer is a promising non-viral method of gene delivery. In our in vitro study we addressed open questions about this multistep process: how electropermeabilization is related to electrotransfer efficiency; the role of DNA electrophoresis for contact and transfer across the membrane; visualization and theoretical analysis of DNA-membrane interaction and its relation to final transfection efficiency; and the differences between plated and suspended cells. Combinations of high-voltage and low-voltage pulses were used. We obtained that electrophoresis is required for the insertion of DNA into the permeabilized membrane. The inserted DNA is slowly transferred into the cytosol, and nuclear entry is a limiting factor for optimal transfection. The quantification and theoretical analysis of the crucial parameters reveals that DNA-membrane interaction (*N_DNA_*) increases with higher DNA concentration or with the addition of electrophoretic LV pulses while transfection efficiency reaches saturation. We explain the differences between the transfection of cell suspensions and plated cells due to the more homogeneous size, shape and movement of suspended cells. Our results suggest that DNA is either translocated through the stable electropores or enters by electo-stimulated endocytosis, possibly dependent on pulse parameters. Understanding of the mechanisms enables the selection of optimal electric protocols for specific applications.

One of the promising methods for delivery of genetic material into a cell is gene electrotransfer, which uses locally delivered electric pulses (electroporation) to transfer DNA into the cell^1^,^2^. The first in vivo gene electrotransfer was demonstrated in the early nineties of the last century by Titomirov^3^ and by several other independent studies^4^,^5^,^6^,^7^,^8^ and has since then been extensively studied^9^,^10^,^11^,^12^. In contrast to viral vector transfer, the use of electric pulses for gene delivery represents a safer method, which is not hampered by concerns of immunogenicity and pathogenicity^13^. Different protocols where designed for in vitro and in vivo applications, either employing short hundreds-of-microsecond pulses^4^, long millisecond pulses^7^,^8^,^14^ or combining high-voltage (10–1000 μs) and low-voltage (10–400 ms) pulses^12^,^15^,^16^,^17^,^18^,^19^. We have recently shown in vitro that^20^,^21^ longer electric pulses are optimal for high transfection efficiency but reduce viability, while shorter pulses enable moderate transfection efficiency and preserve viability. For clinical applications it is also crucial to achieve sufficient transfection efficiency in a given target tissue (e.g. tumor, muscle, skin)^10^,^22^. Gene electrotransfer has in recent years emerged as the most promising non-viral method for delivery of plasmid DNA (pDNA), oligonucleotides and short RNA molecules in gene therapy for a series of conditions like cancer, autoimmune and inflammatory diseases^23^. The first successful clinical trials have already been completed^11^. Recently it has been identified as an ideal method for DNA vaccination for hepatitis, HIV and cancer treatment^24^,^25^,^26^ since electric pulses play a dual role; they enable gene delivery and act as an adjuvant.

However, several papers stress that the mechanisms of gene electrotransfer are still not fully understood^10^,^23^,^27^,^28^. The current description of the process defines several steps: i) electropermeabilization of the cell membrane, ii) contact of the pDNA with the cell membrane (formation of a complex), iii) translocation across the membrane, iv) transfer to and into the nucleus and gene expression^10^,^18^,^19^,^29^,^30^. In addition, new observations of the role of the cytoskeleton and endocytosis have also been published^31^,^32^. From the perspective of clinical applications, all of these steps relate to the barriers which must be overcome for sufficient DNA delivery and expression in the target tissue.

- The first step is cell membrane electropermeabilization, where electric pulses are applied and the transmembrane voltage is induced due to Maxwell-Wagner polarizations. Above a critical (threshold) transmembrane potential (between 0.2–1 V), a high electric field leads to the formation of hydrophilic pores, thus enabling the transfer of molecules into cells^2^. Ions and small molecules enter the cells by free diffusion through pores during and after pulse delivery^33^,^34^, while the transfer of large pDNA is not governed by diffusion, but is a more complex process^15^,^18^,^19^,^29^,^30^,^34^.
- The second step is the interaction of the DNA with the permeabilized cell membrane. It was shown that DNA has to be present in close proximity of the cell membrane at the moment of pulse delivery, enabling contact between the DNA and the electropermeabilized membrane^16^,^17^,^20^,^21^,^29^,^30^,^35^,^36^. The DNA-membrane complex formation was first suggested when the effect of divalent cations^37^,^38^,^39^ on electrotransfection was studied. The complex presents binding to the membrane or partial insertion of DNA in the permeabilized cell membrane^15^,^16^,^19^,^29^,^30^. The first visualization performed by labeling DNA with fluorescent dye TOTO-1^41^ showed complex formation only on the cathodic side of the cell. In addition, within tissues the extracellular matrix represents another barrier which reduces the amount of DNA interacting with the target cells, by hindering the homogeneous distribution of the injected DNA and by decreased DNA mobility during electric pulses^40^,^41^,^42^. The free diffusion of DNA is almost negligible compared to electrophoretic drag, therefore, the selection of sufficiently high and long pulses is important^12^,^16^,^17^,^21^,^40^,^43^.
- The third step is the transfer of DNA into the cells. The process of DNA transfer across the cell membrane has not been directly visualized yet. The DNA enters the cytoplasm several minutes after pulse application^41^,^48^; first the contact of the DNA with the permeabilized cell membrane is formed^15^,^19^,^34^,^44^, then the DNA is either translocated across the cell membrane by an unidentified mechanism^20^,^23^, or alternatively, the DNA enters the cells by electric-field-stimulated endocytosis^32^,^45^,^46^. Recently^32^, it was suggested that endocytosis could play an important role in gene electrotransfer, however, our results do not support this hypothesis^47^.
- The last step for efficient electrotransfer is the intracellular trafficking of the DNA through cytosol and nuclear import. Different cytoplasmic structures hinder DNA mobility inside the cytosol, and foreign DNA is also exposed to DNase activity^48^,^49^. It was demonstrated that^50^ pDNA is probably actively transferred to the nucleus via the tubulin network. Further, during mitosis when the nuclear envelope is disintegrated, the highest electrotransfer efficiency was obtained^51^. It was shown that plasmids containing a nuclear-localization-sequence (NLS) that enables active transport across nuclear pores increased the gene electrotransfer efficiency^50^,^52^,^53^.

In spite of numerous experimental studies, only a few are combined with a theoretical description of DNA-membrane interaction^34^,^37^, the quantification of the number of plasmid DNA^54^ or the DNA mobility in a complex environment such as tissue^40^,^41^,^55^. In this paper we present a systematic in vitro analysis of all the steps of gene electrotransfer. In addition, the differences between cells in a suspension and plated cells are discussed. The most undefined process is the mode of DNA transfer across the cell membrane. We approached this question by different pulse combinations of high-voltage (HV) and low-voltage (LV) pulses (e.g. HV+LV, LV+HV), enabling separate analysis of electropermeabilization and electrophoresis. Our results provide new insights into the processes important for further development of in vitro gene electrotransfer protocols for biotechnological and biomedical applications.

## Methods

### Cell cultures, electroporation buffers and plasmid

Chinese hamster ovary CHO cells (European Collection of Cell Cultures) were grown in F12 HAM (Gibco) supplemented with 1 mM L glutamine, 10% fetal bovine serum (PAA, Austria) and antibiotics at 5% CO_2_ and 37°C. Most of the experiments were performed on cells in the early exponential growth phase (24 h after trypsinization). To evaluate the effect of the stage of the cell culture, cells in the early plato phase (72 h after trypsinization) arrested in the G1 stage of the cell cycle were used. The electroporation buffer was iso-osmolar 10 mM NaH_2_PO_4_/Na_2_HPO_4_, 1 mM MgCl_2_ and 250 mM sucrose, pH = 7.2, while for the visualization of the DNA-membrane interaction with TOTO, we used 10 mM KH_2_PO_4_/K_2_HPO_4_ with the same additives. For all gene electrotransfer experiments, we used plasmid pEGFP-N1 (Clontech Laboratories Inc., Mountain View, CA, USA).

### Electric pulse protocols

Three different pulsing protocols, consisting of either high-voltage HV pulses only, low-voltage LV pulses only, or a combination of both HV and LV pulses (HV-LV combinations) were used for both plated cells and cells in suspensions. To generate electric pulses, a Cliniporator^TM^ (Igea, Italy) and a prototype of the electric pulse generator described in Ref. 19 were used. Parallel wire electrodes were used for the plated cells^18^ and parallel plate electrodes (Eppendorf, Germany) for cell suspension. The distance between the electrodes was 4 mm. All the experiments were repeated at least three times at different dates. In all experiments, the standard HV pulses were 4 × 200 μs pulses, 1 Hz with pulse amplitude *U* = 400 V (applied electric field *E_HV_* = 1 kV/cm), except in experiments where *E* was varied, and the standard LV pulse was 1 × 100 ms, with pulse amplitude 30 V (*E_LV_* = 0.075 kV/cm – LV_30_), except in experiments where higher LV was also used *E_LV_* = 0.137 kV/cm (LV_55_). In the HV+LV protocol, the LV pulse was applied after the HV, with a lag of 20 ms^18^,^19^, while for the LV+HV protocol, the sequence was reversed.

### Cell membrane permeabilization

Cell membrane permeabilization was determined by the uptake of 150 μM propidium iodide (PI) (Invitrogene, Germany), added immediately before electroporation. For each experiment, a negative control - cells not exposed to an electric field, and positive control - cells exposed to 1.8 kV/cm (100% permeabilization) were prepared. The fluorescence intensity was determined 3 minutes after electroporation in a microplate reader (Tecan, Austria) at a 535/617 nm (excitation/emission) wavelength. The percentage of electroporated cells was calculated as the relative fluorescence intensity vs. the positive control^36^.

### Viability

For plated cells, viability was determined by a manual cell count under bright field optics on an inverted microscope (Zeiss 200, Axiovert, Germany) at 20× objective magnification. The cell viability was calculated as the ratio between the number of all cells counted in the treated sample and the number of all cells in the control sample^18^,^47^. For cell suspensions, viability was determined by clonogenic assay. After electroporation, cells were plated in concentrations of 250 cells per 60 mm Petri dish and grown for six days. The colonies were counted and the viability (%) was determined as the ratio between the number of colonies in the treated sample and the number of all cells in the control sample that were not exposed to electric pulses.

### Electrotransfer of plasmid DNA

Plated cells: 5 × 10^4^ cells were seeded in 24 multiwell plates and maintained in culture for 24 h, then the growth media was replaced with a pulsing buffer containing different concentrations of the plasmid DNA (c_DNA_). After a 2–3 min incubation, samples were electroporated, fetal bovine serum (PAA, Austria) was added (37 μl) and the cells were grown for another 24 h in the culture medium. The next day, the electrotransfer efficiency was determined by fluorescent microscopy (Zeiss 200, Axiovert, Germany, at 488/509 nm). At least 7 images were acquired per parameter for each experiment and the percentage transfection (%TR) was determined as a ratio between the fluorescent cells and the total number of cells counted under bright field optics^18^. For HV-LV pulsing protocols, the average maximal fluoresce intensity – *FL^GFP^* [A.U.] was also determined.

Cells in suspension: cell cultures were trypsinized 24 hours before the experiments. On the day of experiment, a cell suspension of 2.5 × 10^6^ cells/ml was prepared in an electroporation buffer. The optimal c_DNA_ was 40 μg/ml, while sub-optimal c_DNA_ were 10 μg/ml and 5 μg/ml. In addition, we also tested c_DNA_ = 100 μg/ml. The electroporation procedure was the same as for plated cells. Cells were plated in 25 cm^2^ culture dishes for 24 hours. The next day, we prepared a cell suspension (1 × 10^6^ cells/ml) in phosphate-buffered saline (PBS) and the GFP expression was measured by flow cytometry with a Coulter EPICS Altra flow cytometer (Beckman Coulter Electronics) and with a CyFlow space flow cytometer (Partec). For each sample, 10000 cells were analyzed. The collected data were analyzed using FlowJo (Tree Star) software. From this percentage of transfected cells and average fluorescence intensity were obtained.

### Visualization of DNA-membrane interaction and plasmid localization in the cytosol

To visualize DNA interaction with the cell membrane, we stained pEGFP-N_1_ with 2.3 × 10^−4^ M TOTO-1 nucleic acid stain (Molecular Probes – Invitrogen, Carlsbad, California, USA) as described in Ref. 29. Cells (1 × 10^5^ cells/ml) were plated in a Labtech chamber for 1 h in a cell culture medium. Then electroporation media with TOTO-labeled DNA (10 μg/μl and c_DNA_ 2 μg/μl to obtain a detectable fluorescence) was added to the cells and different combinations of HV and LV pulses were applied. The HV amplitude was 1.4 kV/cm. The interaction of the DNA with the membrane was determined by fluorescent microscopy (Zeiss 200, Axiovert, Germany) at 100× objective magnification at 514/533 nm. The TOTO fluorescence intensity (FL^TOTO^) profiles were analyzed (MetaMorph) and the average maximal fluoresce intensity – *FL^TOTO^* [A.U.] was obtained from recorded images (at least 5 images per parameter for each experiment). For localization of the plasmid in the cytosol, the plasmid was labeled with rhodamin dye (TM-Rhodamin, Mirus, USA), observed at 100× objective magnification.

### Stage of the cell culture

Gene electrotransfer was performed in cell suspensions trypsinized 24 h before the experiment, or on cells cultured for 72 h, to obtain confluent cell cultures in the early plato phase arrested in the G1 phase of the cell cycle. Three independent experiments were performed.

### Analytical calculations

#### Calculation of the permeabilized surface of the cell membrane

If a cell is exposed to an external electric field *E*, a transmembrane voltage *U_m_* is induced on the cell membrane. When *U_m_* exceeds the threshold voltage *U_c_*, then the part of the cell membrane where |*U_m_*| > *U_c_* is permeabilized^2^. For a spherical cell of radius *R*, a well-known form of *U_m_* for physiological conditions^34^,^56^ is valid:
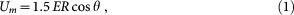
where *θ* is the angle that defines the point on the membrane with respect to the field direction. The above equation is valid for spherical cells, while for spheroidal cells a similar equation is valid taking into account also the shape of the cells^57^. One can define the critical angle *θ*_c_, as the angle where the transmembrane voltage equals the critical voltage:
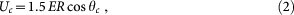
while the critical electric field *E_c_* is defined as *E* where *θ*_c_ = 0, therefore: *E_c_ = U_c_*/*1.5R*. From this, the total area exposed to an above-threshold transmembrane voltage – the permeabilized area – can be obtained:
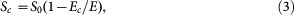
where *S_0_* is total surface of the cell membrane.

#### Analysis of DNA electrophoresis during HV and LV pulses

Electrophoresis is one of the mechanisms that was shown to be important for the efficient delivery of DNA molecules into cells by electric pulses^15^,^16^, especially when plasmid concentration is relatively low^18^,^19^. We present the calculation of the average traveled distance - *L* of a pDNA in aqueous solutions, and from this we estimate the number of DNA molecules available for contact with the permeabilized cell membrane (*N_DNA_*) for our in vitro conditions. We have followed the derivation of Zaharoff and Yuan^41^. Briefly, the electrophoretic force acts on the negatively charged DNA molecules and drags it toward the cathodic side of the cell membrane: *F* = *e_eff_* *E*, where the effective charge (*e_eff_*) depends on the ionic strength of the solution and the length of the plasmid (*e_eff_* = 0.066 e per base pair × 4.7 kbp for pEGFP plasmid). We can use the approximation that during the electric pulses we have a steady-state condition^41^, thus:
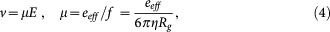
where *v* is velocity of the molecular movement, μ is electrophoretic mobility and *f* is the Stokes' frictional drag, *R_g_* ≈ 100 nm is the approximate radius of gyration and *η* = 0.01 g cm^−1^ s^−1^ is the viscosity of the medium. In all calculations, *E* represents the homogeneous electric field strength, which is justified for our geometry of two parallel electrodes. The distance *L* traveled due to electrophoresis can be thus calculated from the total duration of the electric pulses *t_E_*:
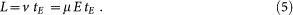
Here we have to stress that the mobility *μ* of a DNA molecule during electric pulses is a complex function of the electric field strength, due to several effects like the elongation and orientation of DNA molecules in the electric field. Therefore, mobility is not the same for HV or LV pulses, however, as already shown^41^, we can use the approximation of constant *μ* for our conditions. For a 4.7 kbp supercoiled pEGFP, *μ* is similar as in Ref. 34: *μ* = 1.5 × 10^4^ μm^2^/Vs; where 4.3 kbp pDNA was used, a similar value can be obtained directly from Eq. 4.

## Results

### Electropermeabilization, electrotransfection and cell viability for different electric field strengths

**Figure 1:**
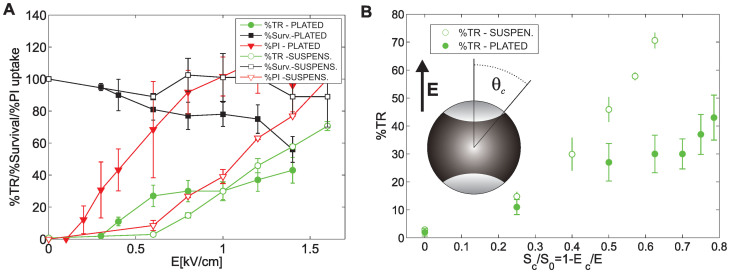
(A) Effect of electric field strength on the percentage transfection of CHO cells (%TR), viability (%Survival) and electropermeabilization (%PI uptake) for 4 × 200 μs pulses (HV) and 1 Hz repetition frequency. Results are shown for plated cells (closed symbols) and cells in a suspension (open symbols) and are presented as mean ± standard error of at least three independent experiments. (B) Dependence of %TR on the fraction of permeabilized membrane area S_c_/S_0_ as given in Eq. 3, for plated cells and cells in a suspension. The bright shaded surface of the spherical cell represents the permeabilized cell membrane - S_c_.

In Fig. 1A, we present the effect of the electric field strength (*E*) on the electropermeabilization (%PI positive cells), percentage transfection (%TR) and survival (%viable cells) for a train of four high-voltage pulses (HV) of 200 μs duration and 1 Hz repetition frequency for plated cells and for cells in a suspension. Electropermeabilization increased above a certain electric field threshold (*E_c_*) and electrotransfer occurred only above the *E_c_*. In a suspension, much higher maximal %TR was achieved (70%) compared to plated cells (40%), even though in both cases the maximal electropermeabilization was reached (around 100%). Plated cells were more affected by electric pulses in terms of cell survival, namely viability dropped to 55%, while for cells in suspension it was maintained at approximately 90% at the highest electric fields (>1.4 kV/cm). In Fig. 1B, the %TR is presented with respect to the electropermeabilized membrane area (Eq. 3).

### Role of electrophoresis - effect of plasmid concentration on electrotransfer efficiency

We studied electropermeabilization of the cell membrane, electrophoresis and DNA–membrane interaction by applying combinations of HV and LV pulses (HV, LV, HV+LV, LV+HV).

#### Electropermeabilization for different HV and LV pulses

In order to separate the electrophoretic effect of LV pulses from electropermeabilization, we determined the PI uptake for different combinations of HV and LV pulses on plated cells and two different pulse amplitudes for LV pulses (30 V and 55 V). We obtained a nearly 100% electropermeabilization for the HV, HV+LV_30_ and HV+LV_55_ pulsing protocols. No statistically significant PI uptake was obtained with the LV_30_ pulse, while for the LV_55_ pulse, PI uptake was 5%. A higher percentage transfection (%TR) was observed for both combinations of HV+LV protocols (HV+ LV_33_ and HV+ LV_55_) and approximately 2% of cells were transfected with only the LV_55_ pulse (results not shown). Therefore, only electrophoretic non-permeabilizing LV_30_ pulses were used in further experiments with LV pulse.

#### The effect of plasmid concentrations on the electrotransfection efficiency for different HV and LV pulses – the role of electrophoresis

**Figure 2:**
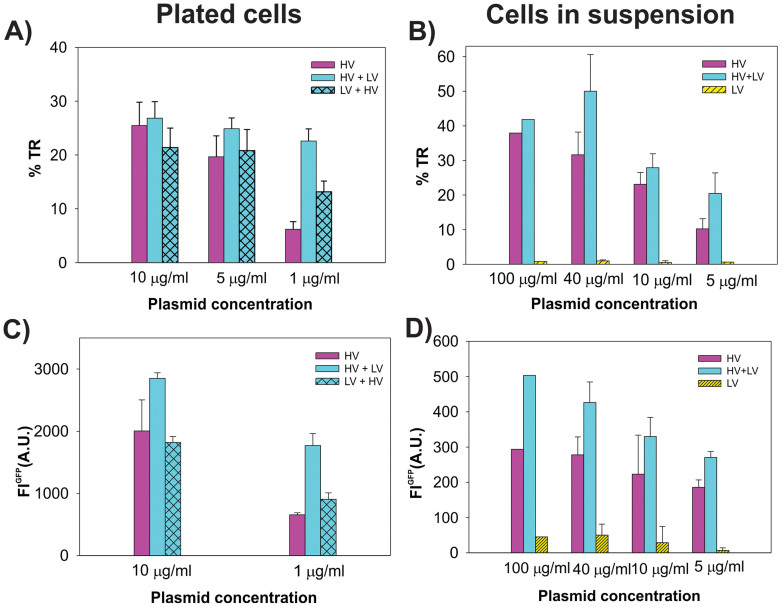
Effect of different HV-LV protocols on the electrotransfer efficiency for different plasmid concentrations. Panels A and C plated cells, B and D cells in a suspension, % TR (A and B) and average maximal fluorescence intensity in A.U. Pulse parameters were: HV pulses (4 × 200 μs), 1 Hz and 1 × 100 ms LV *E_HV_* = 1 kV/cm (400 V) and *E_LV_* = 0.075 kV/cm. The results are presented as mean ± standard error of at least three independent experiments.

In Fig. 2 the effect of HV and LV pulses on the gene electrotransfer efficiency (%TR and fluorescence intensity of FL^GFP^) for different c_DNA_ for cells in suspension and plated cells are presented. For cells in suspension (Figs. 1 B and D), the %TR and mean fluorescence intensity FL^GFP^ obtained by flow cytometry are shown. We obtained that at c_DNA_ = 100 μg/ml, no increase in %TR was obtained for HV+LV pulses compared to HV only (Fig. 2B). For all lower plasmid concentrations (c_DNA_ = 40, 10 and 5 μg/ml), an increase was obtained for HV+LV compared to HV pulses (statistically significant only at 5 μg/ml, P = 0.044). For LV pulses only, a negligible GFP expression was obtained. Similar relationships among the pulsing protocols were obtained when analyzing the mean fluorescence intensity FL^GFP^ (Fig. 2D). Again for all concentrations – up to 40 μg/ml, the HV+LV protocol led to a higher expression of GFP. Also at c_DNA_ = 100 μg/ml, there appears to be a different increase of FL^GFP^ for HV+LV vs. HV pulses only.

In Figs. 2A and C, the results for plated cells are shown, where the percentage transfection (%TR) and fluorescence intensity (FL^GFP^) were determined by fluorescence microscopy. For HV pulses, the %TR was directly dependent on the plasmid concentration; at c_DNA_ = 10 μg/ml, the % TR was 25%, which dropped to only 6% at c_DNA_ = 1 μg/ml. The HV+LV at c_DNA_ = 1 μg/ml lead to a significant increase (P < 0.001) in the percentage transfection (23%) compared to HV pules only (6%). At 5–10 μg/ml plasmid concentrations, the LV pulse did not significantly affect the %TR (P > 0.05). It is important to note that the optimal plasmid concentration for plated cells was 10 μg/ml, while sub-optimal c_DNA_ were 5 μg/ml and 1 μg/ml.

To further investigate the role of electrophoresis on plated cells, we used an additional pulsing protocol consisting of LV pulse applied before the HV pulses (LV+HV). The LV+HV protocol at c_DNA_ = 1 μg/ml significantly increased the %TR cells compared to HV (P = 0.043), but was still less effective than the HV+LV protocol. With only the LV pulse, a negligible number of transfected cells (less than 1%) was obtained. The average maximal fluorescence intensity FL^GFP^ (Fig. 2C) was highest for HV+LV, compared to the HV (P = 0.026) and LV+HV protocols. The increase of FL^GFP^ for the LV+HV vs. the HV protocol was obtained at c_DNA_ = 1 μg/ml, but it was not statistically significant (P > 0.05), while at c_DNA_ = 10 μg/ml, all three pulsing protocols lead to a similar %TR.

### Visualization of DNA-cell membrane interaction and internalization into cytosol

**Figure 3:**
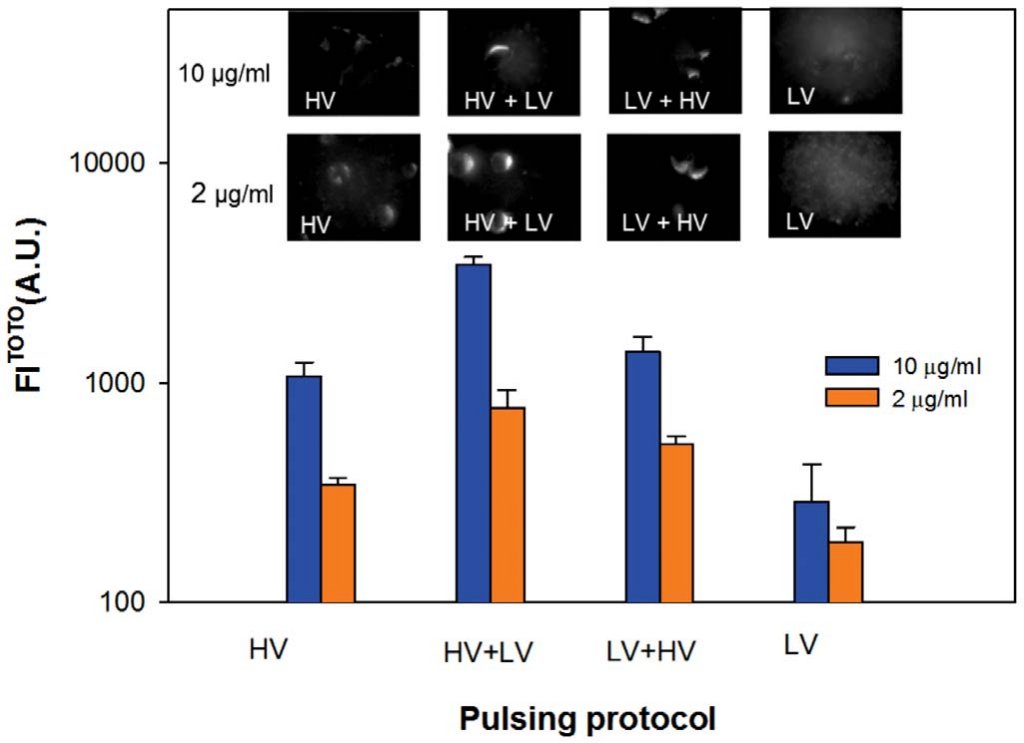
DNA – membrane interaction for different HV-LV protocols for *c_DNA_* = 10 μg/ml and 2 μg/ml. The average maximal fluorescence intensity FL^TOTO^ is presented as an average ± standard error; the representative images for HV, HV+LV, LV+HV and LV pulses are shown on top. Please note that the scale of fluorescence intensity in the micrographs is adjusted between Min = 100 and Max = 700 A.U. in images with *c_DNA_* = 10 μg/μl, and between Min = 100 and Max = 300 A.U. for *c_DNA_* = 2 μg/ml. Immediately after the labeled plasmid was added to the cells, different combinations of HV (4 × 200 μs, *E_HV_* = 1.4 kV/cm, 1 Hz) and LV pulses (1 × 100 ms, *E_LV_* = 0.137 kV/cm) were applied.

Direct visualization of the DNA-membrane interaction was performed by TOTO-1 labeled pDNA for different HV-LV protocols and different c_DNA_. For all protocols, DNA interaction with the cell membrane facing the cathode was observed, since DNA is negatively charged. Fluorescent images of treated cells exposed to HV, HV+LV, LV+HV and LV pulses are shown in Fig. 3 A. The images were acquired 2–5 min after pulsation. The fluorescence intensity FL^TOTO^ corresponds to the amount of TOTO-labeled DNA interacting with the permeabilized cell membrane. Along the membrane, spots of high fluorescence intensity were observed for all pulsing protocols.

The values of FL^TOTO^ (A.U.) were calculated from all recorded images for each pulsing protocol. For HV+LV pulses, an approximately 3× increase in FL^TOTO^ was observed compared to HV pulses only for c_DNA_ = 10 μg/ml and an approximately 2× increase of c_DNA_ = 2 μg/ml (Fig. 3). On average, the FL^TOTO^ of LV+HV was slightly higher compared to HV pulses, but significantly smaller than for the HV+LV protocol. No interaction (the fluorescence intensity was similar to the background) was detected for the non-permeabilizing LV pulse alone. On average, more DNA interaction with the cell membrane and higher FL^TOTO^ was observed for higher amounts of TOTO-labeled DNA (c_DNA_ = 10 μg/ml) compared to sub-optimal c_DNA_ = 1 μg/ml. Further, the entry of pDNA into the cytosol was visualized by rhodamin-labeled DNA. After 10–15 minutes, labeled pDNA was observed inside the cytosol (see Discussion).

### DNA entry into the nucleus

The last step and barrier for the successful electrotransfer of DNA is the nuclear import of pDNA. We designed additional experiments using cells in the exponential and plato stage of growth in order to compare the efficiency of gene electrotransfer of cells in the exponential vs. plato phase of cell culture. We obtained a significantly lower percentage transfection %TR for cells in the plato phase (15.5% ± 1.1) compared to cells in the exponential phase (55.7% ± 11.6) for both the HV and HV+LV (17.3 ± 1.4 plato vs. 52.2 ± 10.2 exponential phase) pulsing protocols as shown in Table 1. We should also stress here that our standard experimental protocol was performed on cells in the exponential phase of cell culture, which is also a standard protocol.

### Theoretical analysis

In the following two subsections we: i) calculate the permeabilized surface area of the cell and ii) analyze electrophoresis for different HV-LV pulses and calculate the *N_DNA_* – the number of DNA molecules available for contact with the permeabilized surface.

#### Calculation of the permeabilized surface of the cell membrane

The permeabilized surface *S*_c_ of the cell membrane depends on the applied field *E* and the threshold field *E_c_*^58^: 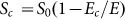 where *S_0_* is total surface of the cell membrane. Thus *E* determines the area of the membrane which is permeabilized – *S*_c_(*E*), and consequently the electrotransfer efficiency. In Fig. 1B, dependence of the % of transfected cells on a normalized permeabilized membrane surface area *S_c_*/*S_0_* (Eq. 3) is shown. For plated cells, an approximately linear dependency on the permeabilized surface was obtained, while for cells in a suspension, a non-linear increase of *S*_c_ with *E* was observed.

#### Analysis of DNA electrophoresis during HV and LV pulses and estimations of the number of DNA molecules in contact with the permeabilized cell membrane

**Figure 4:**
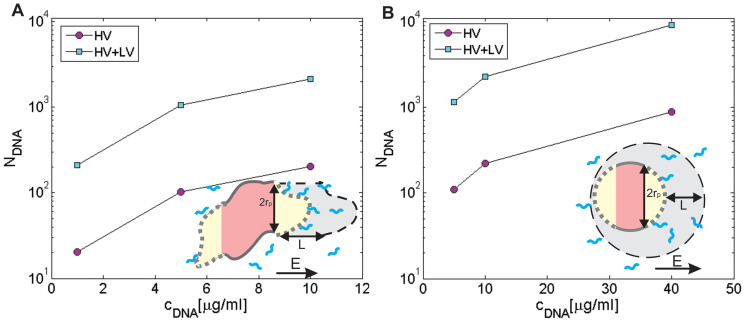
Theoretical analysis of DNA accumulation at the cell membrane due to electrophoretic force. The number of DNA molecules (*N_DNA_*) available for contact with the permeabilized part of the cell membrane for different plasmid *c_DNA_* for plated cells (A) and cells in a suspension (B) are shown for HV and HV+LV pulses (corresponding transfection efficiencies are shown in Fig 1). The schematic representation shows the calculation of *N_DNA_*, where *L* is the distance traveled due to electrophoresis and *r_p_* is the radius of the permeabilized membrane (dotted, yellow area) for plated cells (A) and cells in a suspension (B). The gray shaded region represents the volume *V* from which DNA molecules are brought in contact with the cell membrane. The strength and length of pulses determine the distance *L* from which DNA can access the cell membrane (gray) and *E* determines the area of the membrane which is electropermeabilized.

The traveled distance (*L*) of pDNA during HV and LV pulses due to the electrophoretic force can be obtained from Eq. 5 (see Methods): *L* = *μ E t*_E_. For a single LV pulse (1 × 100 ms, 75 V/cm) we obtain *L_LV_* ≈ 11 μm, *L_HV_* ≈ 1.2 μm and *L_HV+LV_* = 12.2 μm. Thus, for HV+LV pulses the traveled distance is approximately 10× longer compared to HV pulses only (4 × 200 μs, 1 kV/cm). If we assume that the electrophoretic force drags the negatively charged DNA molecules that are at a distance less that *L* from a cathodic site of a cell, we can estimate the number of DNA molecules – *N_DNA_* in the volume *V*, which are available for contact with the permeabilized part of the cell membrane. The results of the calculations of *N_DNA_* for different pulsing protocols are presented in Fig. 4. Thus, if the *c_DNA_* is sub-optimal, it is crucial that the DNA is electrophoretically dragged toward the membrane. For plated CHO cells we obtained the following equation for the volume *V*, from which the DNA molecules are dragged toward the permeabilized part of the cell membrane:
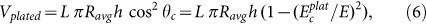
where *R_avg_* is average radius of the plated CHO cells^36^ and the height of a cell is *h* = 4 μm. For plated cells the equation for the induced transmembrane potential for spheroidal cells is valid^57^, however, since the cells are oriented randomly, the average long radius *R_avg_* is a valid approximation, since cells oriented with the long axis in parallel with *E* are first electroporated. For cells in a suspension that are of spherical shapes, the corresponding volume is:
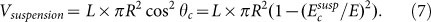
From this we can estimate the *N_DNA_* available for contact formation for different *c_DNA_*:
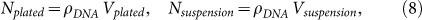
where *ρ_DNA_* is the number density (*ρ_DNA_* = *c_DNA_N_A_/Mr_DNA_*) of DNA for a given plasmid concentration. Thus, from Eqs. 6–8 we can calculate the *N_DNA_* in contact with the permeabilized cell membrane as shown in Fig. 4. The *N_DNA_* is directly proportional to *L* and consequently is approx. 10× lower for HV pulses compared to HV+LV pulses (Fig. 4). From our results we can estimate that for efficient transfection in vitro, several tens of molecules have to be in contact with the cell membrane, while for a smaller *N_DNA_* < 10 the transfection is very low (Figs. 2 and 4).

For cells in a suspension (Fig. 4B), it can be seen that in general a similar relation is obtained: approx. 10× more *N_DNA_* are in contact with the membrane obtained for HV+LV vs. HV pulses, which enables more DNA copies to be transferred into the cytosol, leading to a higher fluorescence intensity in agreement with experimental observations for low plasmid concentrations (Fig. 2D). Thus, the strength and length of the electric pulses determine the distance *L* from which the DNA can access the cell and *E* determines the area of the membrane which is electropermeabilized -*S_c_*. It is also clear that since *N_DNA_* linearly increases with *c_DNA_* and that probability of transfection directly depends on *N_DNA_*, there is a very strong correlation between the %TR and *N_DNA_* until saturation is reached, and the %TR does not increase for higher *N_DNA_*.

When comparing plated and cells in a suspension, we observed that in order to reach comparable transfection efficiencies, higher pDNA concentrations had to be used for cells in a suspension. Also, due to smaller sizes, the threshold for electrotransfer was reached at a higher *E* for cells in suspension compared to plated cells as shown in Ref. 36. Here we extend this study also to HV-LV pulsing combinations. Obviously, electrotransfer of plated cells behaves to some extent differently than with cells in a suspension. The saturation of the %TR for plated cells is already reached at 10 μg/ml compared to cells in suspension, where the highest %TR is reached at 40 μg/ml. Also, *c_DNA_* has to be higher for suspensions compared to plated cells. Moreover, in a suspension, a much higher overall maximal %TR can be obtained, both for CHO (up to 70%) and B16 cells (45%) compared to plated cells (38% for CHO and 25% for B16 cells), and also saturation is reached at a higher *N_DNA_* for cells in a suspension. The results for B16 cells are presented in Ref. 36.

#### Calculation of electric energy

One hypothesis can be that the electric energy needed for DNA interaction with the membrane (complex formation)^29^, is the crucial parameter for electrotransfection, since there exists an energy barrier between the negatively charged DNA and the negatively charged cell membrane. In the most simplified case, we can assume that the electric energy of the pulses *W_e_* equals the work of the electrophoretic force *A_e_* = *F_e_* × *L*. Since *L* = *μ E* *t_E_* we obtain:
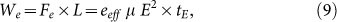
where *E* is the applied electric field strength and *t_E_* is total time duration of all pulses. We can thus estimate *W_e_* of our standard HV (4 × 200 μs, 1 kV/cm), LV (1 × 100 ms, 0.075 kV/cm) and combined HV+LV pulses:
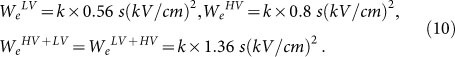
where *k* = e*_eff_ μ.*

#### The relation between electropermeabilization, electrophoresis, DNA-membrane interaction and GFP expression

**Figure 5:**
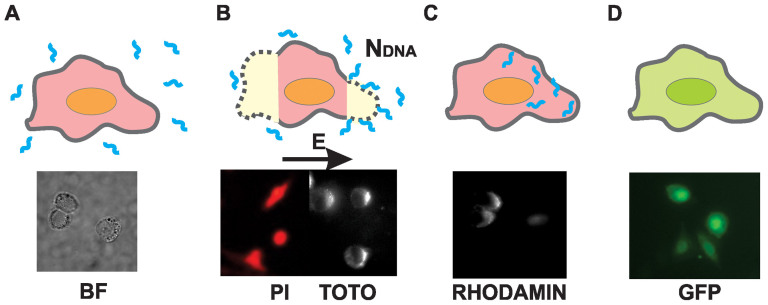
Different steps of gene electrotransfer. (A) DNA is added to the electroporation buffer, (B) electropermeabilization of the cell membrane and DNA contact/insertion with/into the membrane, (C) transfer across the membrane and into the nucleus, (D) gene expression. Corresponding fluorescence images below: electropermeabilization (PI), interaction of DNA with the membrane (TOTO), transfer into cytoplasm (RHODAMIN) and gene expression (GFP).

In Table 2 we summarize the main results related to the different steps (Fig. 5) of electrotransfection. Electropermeabilization was quantified by the %PI of positive cells, DNA-membrane interaction by the analysis of the fluorescence intensity of TOTO-labeled pDNA (FL^TOTO^), gene expression by the fluorescence intensity of GFP (FL^GFP^) and the percentage transfection (%TR), viability by the percentage of survived cells (%Survival), electric energy (*W_e_*) was calculated as defined in Eq. 10 and the number of DNA molecules available for contact with the permeabilized surface (*N_DNA_*) was obtained from Eqs. 7 and 8.

No interaction was observed when only a non-permeabilizing LV_30_ pulse was applied. The detected TOTO fluorescence intensity FL^TOTO^ can be directly related to the *N_DNA_* (Table 2), since the number of DNA molecules interacting with the permeabilized membrane is directly proportional to the plasmid concentration (Fig. 3). The HV+LV protocol yielded the highest FL^TOTO^ followed by the LV+HV and HV protocols for all *c_DNA_*, in contrast to the %TR, where at optimal *c_DNA_* the %TR was similar for all protocols. When we analyze the DNA-membrane interaction (*FL^TOTO^*) in relation to the *N_DNA_* we can see that there is a direct relation between the theoretically calculated number of DNA molecules and *FL^TOTO^*, however, the DNA-membrane interaction is only one of several steps, and other factors such as DNA stability in the cytosol and cell viability are also crucial.

Another aspect is also how the fluorescence intensity of GFP (*FL^GFP^*) is related to the *N_DNA_*. Indeed, HV+LV pulses consistently resulted in a higher *FL^GFP^* compared to HV pulses only, for both plated cells and cells in a suspension (Fig. 2) for all plasmid concentrations. This is also in agreement with other studies (6,14), where for relatively long pulses (8 × 5 ms) *FL^GFP^* can be increased even if the maximal %TR is obtained. However, a statistically significant increase of FL^GFP^ for HV+LV pulses compared to HV was obtained only at the lowest *c_DNA_*, while at the highest *c_DNA_* the difference in FL^GFP^ between the two pulsing protocols was not statistically significant, which also suggests that at some point saturation is reached.

## Discussion

Altogether our results confirm that electropermeabilization is a crucial step for efficient gene delivery, in accordance with other reports^10^,^30^,^34^,^35^,^36^,^59^. This was further confirmed by theoretical analyses of the %TR dependence on the fraction of permeabilized membrane surface *S_c_/S_0_*, where approx. linear dependency was obtained^47^. However, electrotransfection is a complex process and for cells in suspension the experimental values of %TR deviate from the linear curve (see Fig. 1B); thus, the assumption that *S_c_/S_0_* directly correlates with %TR can only be a first approximation.

Further, electrophoresis of the pDNA in an electric field is also an important factor. In order to analyze electropermeabilization and electrophoresis, we used different HV-LV pulsing protocols and different pDNA concentrations (*c_DNA_*). We show that HV+LV pulsing protocols were more efficient in terms of percentage transfection (%TR) and fluorescence intensity (FL^GFP^) compared to HV pulses only, especially for low *c_DNA_*. For higher *c_DNA_*, saturation in terms of maximal %TR was obtained while FL^GFP^ still increased for high *c_DNA_* (see Fig. 2). For in vivo applications, it is important to also analyze the effect of the amplitude of the LV pulse on gene electrotransfer^24^. We obtained that if LV is above the permeabilization threshold (e.g. LV_55_), a high %TR for HV+LV pulses can be attributed both to electrophoresis and to increased electropermeabilization due to the LV pulse, while for sub-threshold low-voltage pulses (LV_30_), LV has only an electrophoretic role.

We extended our previous studies on plated cells and cells in a suspension^18^,^19^,^36^ and analyzed the observed differences. This is relevant since cells in suspension are most often used in vitro, while plated cell are closer to in vivo conditions. A theoretical analysis is presented in Fig. 4, where the results of calculations of the *N_DNA_* (the number of DNA molecules that are available for contact with the permeabilized cell membrane) for different pulsing protocols are shown.

**Figure 6:**
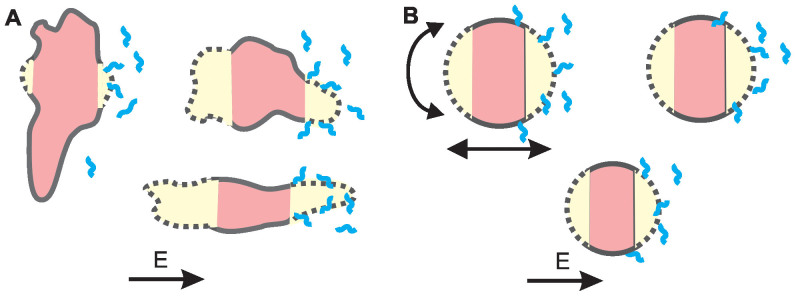
Schematic representation of electrotransfection of plated cells (A) and cells in a suspension (B). Cells in a suspension are spherical and with a narrow distribution of sizes, while plated cells have large variations in size, orientation and shape. Consequently, for plated cells it is very difficult to achieve pulses that would enable optimal electropermeabilization (yellow) and electrotransfer of all cells, in contrast to cells in suspension where this is possible. Furthermore, cells in a suspension rotate and move during pulses, thus a higher *N_DNA_* is needed and therefore a higher *c_DNA_* must be used compared to plated cells.

Based on all the results of HV-LV pulses, we confirmed the hypothesis that in conditions where we reach saturation (high *N_DNA_*), an additional electrophoretic LV pulse does not increase the %TR, while the GFP expression (FL^GFP^) can still be increased (see Figs. 2 and 4). Theoretical analysis showed that the strength and length of the electric pulses determine the distance *L* from which DNA can access the cell, and *E* also determines the area (*S_c_*) of the membrane which is electropermeabilized. We could adequately explain the differences in transfection for different *c_DNA_*, but some data still seemed puzzling. The observed differences in electrotransfection between plated cells and cells in a suspension can be explained by several specific characteristics of the attached cells and suspended cells. This is presented in Fig. 6, where a schematic representation of electropermeabilization and electrotransfection of the plated cells (6A) and cells in a suspension (6B) is shown, and can be analyzed as follows:

- different forces acting on cells in a suspension during pulse delivery, like fluid flow and electrophoretic movement due to the cells' negative surface charge, lead to the displacement and rotation of the cells. This leads to permeabilization of a larger area of the cell membrane (see Fig. 6B), which makes more binding sites available for DNA and increases the maximal *N_DNA_* as shown in Fig. 4B, thus allowing more DNA molecules to be transferred. It was shown previously^30^ that cell electropermeabilization and electrotransfer form different sides, enables higher transfection efficiency. Thus, the rotation of suspended cells enables higher transfection (Fig. 6). In addition, for plated cells due to geometry (cells are spread on the surface) smaller surface is available for DNA binding/insertion. This explains why saturation is reached at much higher *N_DNA_* values for cells in a suspension compared to plated cells (see Figs. 2 and 4). It also explains partially why the overall maximal %TR can be much higher in a suspension;
- with a very low *c_DNA_* in a suspension, there is a very low probability of *N_DNA_* insertion into the membrane, since there is only a small number of molecules available for contact, and in combination with the movement of cells (displacement and rotation) in a suspension (Fig. 6B), this leads to a very low overall transfection for lower plasmid concentrations. Therefore, the *c_DNA_* for suspensions has to be increased compared to plated cells, where cells are attached to the surface and all DNA in the vicinity can form a contact with the membrane. For increasing *c_DNA_* and higher *N_DNA_*, the transfection efficiency can be significantly increased in a suspension, and saturation is only reached at very high numbers of *N_DNA_* (~10^3^), as shown in Fig. 4B. In contrast, with plated cells saturation is already reached at much smaller *c_DNA_* and *N_DNA_* (~300), as can be seen in Fig. 4A, since the entire permeabilized surface is already occupied by DNA molecules.
- the overall maximal %TR in a suspension is much higher than in plated cells (Fig. 1), but this is logical if we take into account that cells in a suspension have a very homogenous distribution of sizes. Thus, with optimal pulses most of the cells are permeabilized and remain viable, enabling good electrotransfer efficiency. On the other hand, plated cells are of different sizes and non-spherical shapes, with large variations in the minimal and maximal diameter, and with different orientations (Fig. 6A)^36^,^57^. Therefore, optimal pulses for the transfection of some cells directly lead to the irreversible electroporation or poor transfection of other cells. Thus, saturation for high *N_DNA_* and maximal %TR is limited for plated cells, due also to the non-homogeneous distribution of sizes and shapes.

Further, we can assume two hypothesis regarding the most important parameters relevant for efficient electrotransfer; i) the electric energy of the pulses is the crucial parameter for efficient transfection or ii) the number of DNA molecules available for contact with the permeabilized membrane (*N_DNA_*) is the most relevant parameter. Based on the presented data and theoretical analysis, we can assume that not *W_e_* but the accumulation of DNA molecules at the permeabilized membrane surface (*N_DNA_*) is the most important factor. *N_DNA_* is approximately linearly dependent on the *E*, *t_E_*, *c_DNA_* and permeabilized membrane surface *S_c_*:
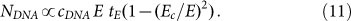
The assumption that the electrotransfer efficiency is proportional to *N_DNA_* is only an approximation, since these are stochastic processes and consequently the probability of electrotransfer is proportional to the *N_DNA_* available for electrotransfer until saturation is reached (explained below). From Table 2, it is clear that *W_e_* could not be the only factor for efficient gene transfer, since the energy of the LV pulse alone is of the same order of magnitude as the energy of HV pulses, while transfection is negligible for the LV pulse. Electric energy also cannot explain the results obtained for the different *c_DNA_*.

On the other hand, *N_DNA_* is the parameter that is directly related with experimental results, especially for sub-optimal pDNA concentrations. It can be seen that the *N_DNA_* for HV+LV pulses (250) for the lowest pDNA concentration (1 μg/ml) on plated cells is similar to the *N_DNA_* for HV pulses only (200) at the highest *c_DNA_* = 10 μg/ml, which is in agreement with similar values of %TR (23% and 25%) obtained experimentally (Table 2). Therefore, %TR is directly related to *N_DNA_* up to the point where saturation is reached, and an increase in *N_DNA_* does not lead to a higher %TR (for plated cells saturation is reached at *N_DNA_* ≅ 250). Consequently, we can explain why at *c_DNA_* = 10 μg/ml, the HV+LV pulsing protocol (*N_DNA_ ≅ 2500*) was similarly effective as the HV protocol (*N_DNA_ ≅ 250*) for plated cells (approx. 25% TR for both protocols). For the LV+HV protocol, this is not so clear since LV is applied before permeabilizing HV pulses, and thus it contributes only to the accumulation but not to the insertion of pDNA into the membrane.

Therefore, the second hypothesis – that the number of DNA molecules interacting with the permeabilized cell membrane is a more relevant parameter – seems more plausible. If we analyze *N_DNA_*, it can be seen that electrophoresis is important for low *c_DNA_*, where the accumulation of DNA at the membrane is a limiting factor (Table 2), while for higher *c_DNA_* the saturation is reached. Therefore, the %TR is directly related to *N_DNA_* for sub-optimal plasmid concentrations, while for higher *c_DNA_* the %TR does not depend directly on *N_DNA_* due to saturation.

If we now summarize the main results in Table 2, we can see that there is a direct relation between the DNA-membrane interaction FL^TOTO^ with the transfection efficiency (FL^GFP^ and %TR) for lower plasmid concentrations. For increased *c_DNA_* (10 μg/ml), saturation is reached in terms of maximal %TR and partially for FL^GFP^. Therefore, at some point even if *N_DNA_* interacting with the membrane FL^TOTO^ is increased, the %TR cannot be increased. This clearly shows on one hand that the transfection efficiency is directly related to electropermeabilization, and that it is a stochastic process in which more DNA in contact with the permeabilized part of the membrane enables more molecules to enter. But at some point, there is saturation as the process becomes similar to a chemical process, where there is a limited number of binding sites, naturally leading to saturation.

Another important aspect is also the physiological state of the cell and its viability, which limits transfection efficiency; namely the total yield of transfected cells is lowered since some cells are very effectively transfected while others are in poor physiological condition or die due to extensive membrane damage and the loss of cell homeostasis. Thus, depending on the type of application, the selection of the specific pulsing protocol depends on whether high yield or high loading is needed. Thus, we proposed^21^ that in in vitro conditions for a high number of copies transferred, it is advisable to use long-duration millisecond pulsing protocols or a combination of HV+LV pulses^10^,^35^,^43^, while for certain biomedical and biotechnological applications where the total yield of transfected cells and/or preserved viability is crucial (e.g. immuno-gene therapy)^9^, short-duration pulse protocols are more optimal. We have to stress, however, that in vivo the differences between tissue properties also determine the choice of optimal electric pulse parameters.

**Figure 7:**
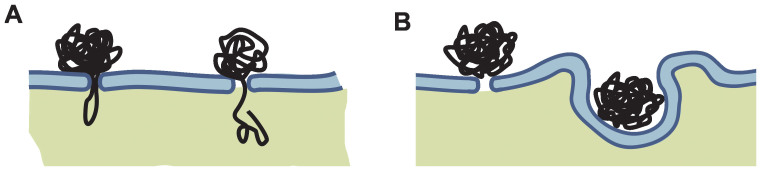
Two possible hypotheses of DNA entry into the cytoplasm. (A) DNA is inserted into the permeabilized cell membrane during electric pulses and is translocated inside the cell by a slow process after pulse delivery; (B) DNA interacts with the permeabilized membrane and is consequently endocytosed.

Further, we addressed another open question: how does DNA cross the cell membrane and enter the cytosol? There are two main hypotheses of DNA entry, as schematically presented in Fig. 7 above. The first hypothesis suggests that DNA is first inserted into the permeabilized cell membrane and is then transferred into the cytosol by some unknown mechanism^15^,^16^,^19^,^60^ (Fig. 7A). Alternatively, the second hypothesis assumes that DNA-membrane complex formation, in combination with exposure to an electric field, triggers endocytotic invagination of the cell membrane (Fig. 7B), followed by the transfer of DNA into the cytosol. However, endocytotic entry has thus far been directly confirmed only in a few reports^32^,^46^. No direct observation of DNA transfer across the membrane has been observed; direct visualization only showed^21^,^29^,^30^ that formation of a stable DNA-membrane complex occurs on a time scale of 1 s after EP and that DNA enters in minutes after pulse application^29^,^30^. Our results confirmed that DNA enters the cytosol in minutes after pulse delivery as shown in Fig. 5C. However, when we designed a separated study to analyze the role of electrostimulated endocytosis in gene electrotransfer, we have not confirmed this hypothesis^47^ for our pulsing protocol. Visualization of endocytotic vesicles after the application of our HV pulses showed that the level of endocytosis did not increase above the threshold electric field for electrotransfer, suggesting that electro-endocytosis is not the dominant mechanism for electrotransfer with this protocol. Also in studies^21^,^29^,^30^ where TOTO-labeled pDNA was used for analysis of the interaction with the membrane, no endocytotic uptake was observed – in our experiments fluorescence spots were also observed 15 min after electroporation, only on the cell membrane (results not shown) and not in the cytoplasm.

Further, by using HV+LV and the reversed order of LV+HV pulses, we could indirectly analyze the mechanism of how DNA enters the cytosol. However, since a significantly higher transfection efficiency and DNA-membrane interaction was observed for HV+LV compared to the LV+HV protocol (Table 2) at sub-optimal 1 μg/ml *c_DNA_*, we propose that the LV pulse applied after HV pulses is crucial for DNA insertion and/or translocation across the cell membrane, and not only for accumulation of DNA at the cell membrane surface. This further supports the hypothesis that DNA is first inserted into the permeabilized membrane and later enters the cytoplasm via translocation across the membrane pores (Fig. 7A).

Taking into account all results of our and other studies, we propose that DNA insertion into the permeabilized membrane during electric pulses is a first and crucial step for later DNA entry via either translocation or electroendocytosis (Fig. 7B), and that the way of DNA entry might also depend on the specific pulsing protocol. The process probably involves DNA interaction with the permeabilized cell membrane and not only the simple contact of the DNA with the membrane. The possible mechanism of DNA translocation through electropores could be a mechanism similar to the Brownian ratchet, which was described for nucleotide translocation through nanopores due to entropic forces^19^,^61^,^62^. So far the most developed theoretical description of electroporation is a model of formation of aqueous pores in the membrane^2^, and a very consistent and experimentally verified theoretical framework was also presented describing pore formation and resealing^63^. However, for electrotransfection no such theoretical description exists, thus the mechanism of DNA entry is still an open question.

The last step for successful electrotransfer is the entry of pDNA into the nucleus, since the nuclear envelope is not permeabilized by the standard electroporation pulses commonly used. The majority of plasmids including pDNA, used in our study have the NLS sequence for enhanced transport into the nucleus encoded in SV 40 DLS^31^,^52^. In spite of the NLS sequence, better electrotransfer efficiency is obtained in mitotic cells^49^,^51^, confirmed also in our experiments. We obtained an approx. 3× times higher %TR for cells in an exponential phase compared to cells in a plato phase (arrested in the G1 phase), as shown in Table 1. This is especially important for clinical applications since most of the somatic cells in tissues are not actively dividing. It was shown that plasmid containing a specific NLS designed for a particular target tissue can significantly improve the transfer efficiency^53^.

## Conclusions

In this paper we integrate an experimental and theoretical analysis of the different steps involved in gene electrotransfer in order to gain new insights into the processes involved. We show that the number of DNA molecules in contact with a permeabilized membrane is governed by the electropermeabilized surface, the electrophoretic force and the pDNA concentration. The inserted DNA is slowly transferred into the cytosol in minutes following the pulses. Nuclear entry can be a limiting factor for in vivo application where cells are not actively dividing, while in vitro cells in the exponential growth phase must be used for optimal transfection. We also explain the differences between the electrotransfer efficiency of suspended and plated cells; the later represent a more relevant system for in vivo applications, while cell suspensions enable a higher transfection yield due to the more homogeneous size, shape and movement of suspended cells.

For different HV-LV pulsing protocols, we analyzed the crucial parameters: from DNA interaction with the permeabilized cell membrane (*N_DNA_*) to the observed transfection efficiency (%TR, FL^GFP^), and their mutual relations. We obtained that *N_DNA_* and FL^TOTO^ increase with higher DNA concentrations or with the addition of LV pulses, while %TR and FL^GFP^ both reach saturation. Therefore, direct correlation between interaction and transfection efficiency exists only to a certain point where saturation is reached, due to a limited number of DNA molecules that can interact with the permeabilized surface of the membrane and consequently be transferred.

By understanding the interplay of these parameters, one can design a more optimal electric protocol for a specific application, where either high loading of the plasmid with moderate cell viability or moderate transfection efficiency with preserved viability can be obtained. Further, pDNA concentration is also important: for sub-optimal *c_DNA_* (realistic for in vivo conditions), saturation is not reached, thus electrophoresis plays an important role, while for optimal *c_DNA_* (high *N_DNA_*) a higher transfection yield can be achieved (e.g. in vitro). Finally, as a mechanism of DNA electrotransfer into cells, we propose that after insertion into the permeabilized membrane, DNA is either translocated into the cytoplasm after the pulses by some relatively slow mechanism such as Brownian ratchet, or alternatively it may be transferred by electric-field stimulated endocytosis, or both, where the mechanism probably depends on the choice of pulse parameters.

## Figures and Tables

**Table 1:** Efficiency of gene electrotransfer in terms of % transfected cells (%TR) for CHO cells in a suspension in the exponential growth phase vs. cells in the plato phase for the HV (4 × 200 μs, *E_HV_* = 1.4 kV/cm, 1 Hz) and HV+LV (HV 4 × 200 μs, *E_HV_* = 1.4 kV/cm, LV 1 × 100 ms, *E_LV_* = 0.0375 kV/cm) pulsing protocols. The results are presented as mean ± standard error of at least three independent experiments, *c_DNA_* = 40 μg/μl

%TR PHASE OF CULTURE	HV	HV+LV	LV
**exponential**	55.7 ± 11.6	52.2 ± 10.2	0.3 ± 0.1
**plato**	15.5 ± 1.1	17.3 ± 1.4	0.1 ± 0.0

**Table 2:** Quantification of the process related to the different steps of gene electrotransfer: electropermeabilization – PI uptake (%PI), DNA–membrane interaction represented by the fluorescence intensity of TOTO-labeled DNA (FL^TOTO^), gene expression represented by the fluorescence intensity of GFP (FL^GFP^), percentage of transfection (%TR), viability (%survival) for c_DNA_ = 10 μg/ml, electric energy obtained from Eq. 10 (We), and estimation of DNA accumulation at the cell membrane – the calculation of number of DNA molecules (N_DNA_) interacting with the permeabilized cell membrane calculated from Eqs. 7 and 8 for different pulsing protocols: HV: 4 × 200 μs, 1.0 kV/cm, 1 Hz; LV: 1 × 100 ms, 0.075 kV/cm*. All data are given for plated CHO cells; for better readability only mean values are given

Pulsing protocol	Permea-bilisation	Interaction	Transfection		*Viability*	*El. energy*	*Electro-phoresis*
	%PI	aFL^TOTO^ [A.U.]	FL^GFP^ [A.U.]	%TR	*%Survival*	*W_e_/k = E*^*2*^* × t_E_* ms×(kV/cm)^2^	*N_DNA_*
**HV**							
**c_DNA_ = 1 μg/ml**	82.9%	343	658	6%	82%	0.8	20
**c_DNA_ = 10 μg/ml**		1073	2004	25%			200
**HV+LV**							
**c_DNA_ = 1 μg/ml**	85.9%	772	1769	23%	91%	1.36	250
**c_DNA_ = 10 μg/ml**		3469	2852	27%			2500
**LV+HV**							
**c_DNA_ = 1 μg/ml**	85.9%^b^	527	904	13%	87%	1.36	20–250
**c_DNA_ = 10 μg/ml**		1386	1819	21%			200–2500^b^
**LV**							
**c_DNA_ = 1 μg/ml**	1.5%	187	208	0.9%	78%	0.56	0^d^
**c_DNA_ = 10 μg/ml**		289^c^	258	0.3%			

^a^for observation of DNA–membrane interaction, we used 2 μg/ml DNA and 1.4 kV/cm short-duration pulses instead of 1.0 kV/cm (see the M&M section) and for LV, 0.137 kV/cm instead of 0.075 kV/cm.

^b^since LV is applied before HV, it contributes only to accumulation but not to insertion; the %PI is taken from the HV+LV protocol.

^c^similar to the background value.

^d^the permeabilized surface is zero (see Theoretical section).
